# Bioactive Dietary VDR Ligands Regulate Genes Encoding Biomarkers of Skin Repair That Are Associated with Risk for Psoriasis

**DOI:** 10.3390/nu10020174

**Published:** 2018-02-04

**Authors:** Amitis Karrys, Islam Rady, Roxane-Cherille N. Chamcheu, Marya S. Sabir, Sanchita Mallick, Jean Christopher Chamcheu, Peter W. Jurutka, Mark R. Haussler, G. Kerr Whitfield

**Affiliations:** 1 School of Life Sciences, Arizona State University, Tempe, AZ 85281, USA; tkarrys12@gmail.com; 2 Department of Basic Medical Sciences, University of Arizona College of Medicine–Phoenix, Phoenix, AZ 85004, USA; peter.jurutka@asu.edu (P.W.J.); haussler@email.arizona.edu (M.R.H.); gkw@email.arizona.edu (G.K.W.); 3 Department of Dermatology, School of Medicine and Public Health, University of Wisconsin, Madison, WI 53706, USA; irady@dermatology.wisc.edu (I.R.); roxanechamcheu@gmail.com (R.-C.N.C.); 4 Department of Zoology, Faculty of Science, Al-Azhar University, P.O. Box 11884 Nazr City, Cairo, Egypt; 5 School of Mathematical and Natural Sciences, Arizona State University, Phoenix, AZ 85306, USA; msabir@asu.edu (M.S.S.); smallic5@asu.edu (S.M.); 6 Department of Basic Pharmaceutical Sciences, School of Pharmacy, College of Health & Pharmaceutical Sciences, University of Louisiana at Monroe, 1800 Bienville Drive, Bienville 362, Monroe, LA 71201, USA

**Keywords:** vitamin D receptor, late cornified envelope genes, docosahexaenoic acid, curcumin, epidermis, keratinocytes, psoriasis treatment, nutraceuticals, differentiation, activator protein-1

## Abstract

Treatment with 1,25-dihydroxyvitamin D_3_ (1,25D) improves psoriasis symptoms, possibly by inducing the expression of late cornified envelope (*LCE*)3 genes involved in skin repair. In psoriasis patients, the majority of whom harbor genomic deletion of *LCE3B* and *LCE3C* (*LCE3C_LCE3B-del*), we propose that certain dietary analogues of 1,25D activate the expression of residual *LCE3A/LCE3D/LCE3E* genes to compensate for the loss of *LCE3B*/*LCE3C* in the deletant genotype. Herein, human keratinocytes (HEKn) homozygous for *LCE3C_LCE3B-del* were treated with docosahexaenoic acid (DHA) and curcumin, two low-affinity, nutrient ligands for the vitamin D receptor (VDR). DHA and curcumin induce the expression of *LCE3A*/*LCE3D/LCE3E* mRNAs at concentrations corresponding to their affinity for VDR. Moreover, immunohistochemical quantitation revealed that the treatment of keratinocytes with DHA or curcumin stimulates *LCE3* protein expression, while simultaneously opposing the tumor necrosis factor-alpha (TNFα)-signaled phosphorylation of mitogen activated protein (MAP) kinases, p38 and Jun amino-terminal kinase (JNK), thereby overcoming inflammation biomarkers elicited by TNFα challenge. Finally, DHA and curcumin modulate two transcription factors relevant to psoriatic inflammation, the activator protein-1 factor Jun B and the nuclear receptor NR4A2/NURR1, that is implicated as a mediator of VDR ligand-triggered gene control. These findings provide insights into the mechanism(s) whereby dietary VDR ligands alter inflammatory and barrier functions relevant to skin repair, and may provide a molecular basis for improved treatments for mild/moderate psoriasis.

## 1. Introduction

Psoriasis is a skin disease of largely unknown etiology with an estimated prevalence of over three percent in the United States [1]. Prominent features include overproliferation and incomplete differentiation of epidermal keratinocytes, as well as epidermal inflammation. Chemical analogs of 1,25 dihydroxyvitamin D_3_ (1,25D), the hormonal metabolite of vitamin D, are routinely used as topical agents to treat mild/moderate psoriasis. The success of 1,25D-based agents is presumably related to the reported role of vitamin D in skin biology, in particular its role in regulating keratinocyte proliferation and differentiation, but also its known ability to regulate components of the immune system in skin (reviewed in [2]). In support of the role of vitamin D (or lack thereof) in psoriasis pathogenesis, several studies have associated low vitamin D status with psoriasis (e.g., [3,4]). However, 1,25D analog therapy, even when combined with an anti-inflammatory drug (e.g., betamethasone), is not effective in approximately 40% of patients with mild to moderate psoriasis [5]. Severe cases of psoriasis can be treated effectively with injectable anti-inflammatory agents such as etanercept, adalimumab, infliximab, or secukinumab [6]; however, these agents are not approved for patients with mild/moderate cases [7]. Thus, developing improved topical agents for mild/moderate psoriasis remains an important goal.

The currently accepted paradigm of 1,25D action is that 1,25D, or an analog thereof, binds with high affinity to the nuclear vitamin D receptor (VDR), which then forms a heterodimer with a retinoid X receptor (RXR) isoform on vitamin D-responsive elements (VDREs) in chromosomal DNA, modulating the expression of nearby target genes [8]. Although 1,25D and its analogs are known to improve psoriasis symptoms in many patients, presumably by serving as VDR ligands, the key epidermal genes that are regulated to ameliorate psoriasis are poorly characterized. One approach to enhancing topical therapies for psoriasis is therefore to identify key genes affected by treatment as well as VDR analogs or other ligands that may optimize these effects. Our approach has been to examine those genes for which genetic variations have been shown to confer risk for psoriasis as potential targets for VDR-mediated regulation.

**Figure 1:**
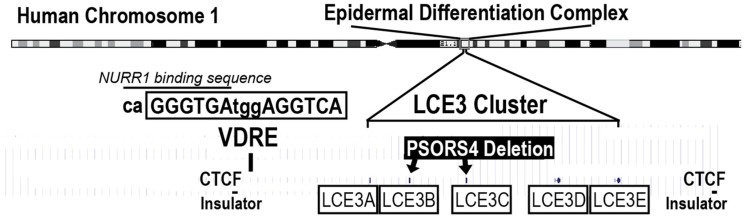
Location of the *LCE3* gene cluster (exact location of each gene indicated by a tiny bars above the boxed LCE3 designation) and the common *PSORS4* gene deletion within the epidermal differentiation complex on human chromosome 1 that also contains other skin genes such as *filaggrin*, *loricrin*, *involucrin*, and *S100A* (not shown). The *PSORS4* deletion eliminates two *LCE3* genes (*LCE3C_LCE3B-del*), but leaves *LCE3A/LCE3D/LCE3E* intact. A putative vitamin D-responsive element (VDRE) is located adjacent to the *LCE3A* gene, but within the boundaries established on either side of the *LCE3* gene cluster by sites for the CCCTC-binding factor (CTCF) that may serve to insulate this gene cluster from other nearby genes. This VDRE has been shown to confer responsiveness to both 1,25D and delphinidin in transfection experiments using a heterologous reporter gene construct [11]. Shown above the VDRE sequence is the location of an overlapping recognition sequence for the nuclear receptor subfamily 4 group A member 2 (NR4A2), also known as NURR1 (see text for explanation).

Psoriasis susceptibility loci (*PSORS)* include the *PSORS4* locus, which is contained within the epidermal differentiation complex (EDC), a large assemblage of over 60 genes expressed during the process of epithelial differentiation (Figure 1). Five closely related late cornified envelope genes (*LCE3A*–*E*) exist in a cluster within the EDC and have been reported to play a role in skin repair [9]. The *PSORS4* risk allele is a deletion of two of these genes, *LCE3C_LCE3B-del*, leading to the hypothesis that the loss of two (of five) *LCE3* genes might reduce the ability of psoriatic lesions to heal [9]. This deletion is very common and studies have indicated that the frequency of *LCE3C_LCE3B-del* is over 50% in many human populations, and is significantly overrepresented in patients with psoriasis, where its frequency increases to 65–75% of the patient population [10].

The endocrine VDR ligand (1,25D), as well as novel VDR ligands delphinidin and cyanidin, have previously been shown to upregulate *LCE3* gene expression to some extent [11,12]. Further, a VDRE has been identified adjacent to the *LCE3A* gene [11] and is not affected by the *LCE3C_LCE3B* deletion (Figure 1). We have proposed that activation by liganded VDR from this VDRE could coordinately upregulate the expression of the *LCE3A/LCE3D/LCE3E* genes to compensate for the *LCE3C_LCE3B* deletion under the assumption that the highly homologous *LCE3* genes have overlapping functions [13]. We have additionally suggested that delphindin and cyanidin represent candidate lead compounds that could be developed into agents to treat mild to moderate psoriasis in a similar manner to how 1,25D was utilized as a lead compound to create therapeutically effective analogs such as calcipotriol [14].

For the current study, we analyzed the ability of two additional bioactive lipids of nutritional origin, namely docosahexaenoic acid (DHA) and curcumin, to upregulate *LCE3* mRNAs as well as proteins. These two compounds were chosen not only for their demonstrated affinity for the vitamin D receptor [15], but also for their reported beneficial effects in skin. Curcumin has been used since ancient times to treat skin inflammation and other ailments, and its anti-inflammatory actions have been studied in the context of various skin conditions, including pruritus, facial photoaging, radiodermatitis, and diabetic microangiopathy (reviewed in [16]). DHA and other omega-3 fatty acids have also been studied for their anti-inflammatory effects, mediated via a variety of mechanisms (reviewed in [17]), including serving as the precursor for the anti-inflammatory molecule resolvin D1 that was recently shown to improve inflammatory symptoms in a mouse imiquimod-induced model of psoriasis [18]. Both DHA [19,20] and curcumin [21,22] have been examined as treatments for psoriasis, but corresponding studies enrolled only a relatively small number of patients and the molecular mechanism(s) accounting for the positive effects observed in a subset of patients were not investigated.

We also examined the ability of 1,25D, curcumin, and DHA to upregulate the expression of two other disease biomarkers, the activator protein-1 factor Jun B and the nuclear receptor NR4A2, to illuminate additional pathways by which 1,25D, curcumin, and/or DHA might improve psoriasis symptoms. Jun B was selected since the *Jun B* gene is localized in psoriasis susceptibility locus 6 (*PSORS6*). Additionally, an inducible epidermal knockout of *Jun B* in adult mice was reported to yield a phenotype possessing the histological and molecular hallmarks of psoriasis [23]. Further, *Jun B* expression, similar to *LCE3* gene expression, is associated with skin repair [24]. NR4A2, also known as NURR1, nuclear receptor of T cells (NOT), or transcriptionally inducible nuclear receptor (TINUR), was selected for study because a putative VDRE located near the *LCE3* gene cluster overlaps with a consensus NR4A2 binding site (Figure 1, caGGGTGA). An additional reason why NR4A2 was of interest is our previously published hypothesis that part of the actions of liganded VDR might be mediated via the upregulation of NR4A2 [25]. Based on the current study, we conclude that both Jun B and NR4A2 may play a role in the action of 1,25D and DHA, and to some extent of curcumin, on skin, but higher doses of ligands are required to demonstrate a statistically significant effect on mRNA levels.

Finally, several literature reports have implied that the effects of DHA in the skin are mediated, not by VDR as we have hypothesized, but rather via peroxisome proliferator-activated receptor (PPAR) isoforms, with PPARδ being the dominant isoform in human skin [26]. To assess whether PPARδ is a major regulator of *LCE* genes, we probed GW501516, a selective ligand for PPARδ, for its ability to upregulate *LCE3* genes in keratinocytes.

## 2. Materials and Methods

### 2.1. Source of Ligands and Reagents

**Figure 2:**
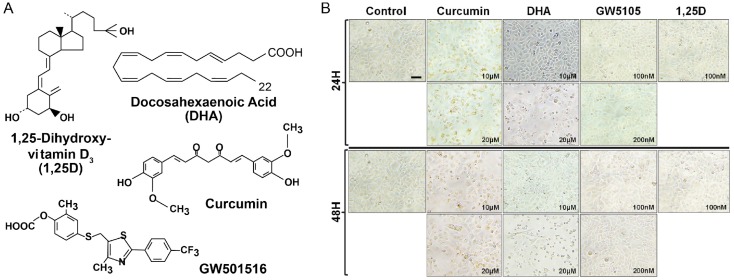
(**A**) Ligands used in the current study. 1,25D is the natural high affinity ligand for vitamin D receptor (VDR). Curcumin and docosahexaenoic acid (DHA) are natural, low-affinity ligands for VDR, and GW501516 is a specific synthetic ligand for peroxisome proliferator-activated receptor (PPAR)δ, with the latter nuclear receptor also having a reported affinity for DHA. (**B**) Morphology of normal human epidermal keratinocytes (NHEKs) treated without ligand (control) or with various ligands at the indicated concentrations and time of incubation. Magnification = 300×.

Crystalline 1,25D was obtained from Roche Diagnostics (Indianapolis, IN, USA). Docosahexaenoic acid was purchased from Sigma Aldrich Corporation (St. Louis, MO, USA). GW510516 was secured from Santa Cruz Biotechnology (Dallas, TX, USA). GW501516 and 1,25D were dissolved in ethanol at 1000 times the concentrations needed for cell culture experiments (1000×) and stored at −20 °C. Curcumin was obtained from Cayman Chemical Co. (Ann Arbor, MI, USA), dissolved in dimethyl sulfoxide (DMSO) at 1000×, and stored at −20 °C. Figure 2A depicts the structures of these ligands. An antibody against NR4A2/NURR1 (LS-C99204) was purchased from LifeSpan BioSciences, Inc. (LSBio, Seattle, WA, USA). Antibodies to LCE3B–E (clone C-14, sc-138974), Jun B (clone N-17X, sc-46X), p-JNK (clone G-7, sc-6254), p-p38 (clone D-8, sc-7973), and filaggrin (clone AKH1, sc-66192) were purchased from Santa Cruz Biotechnology (Santa Cruz, CA, USA). Additional antibodies to LCE3B–E proteins were a kind gift from M. Narita in the laboratory of J. Shalkwijk [27]. Alexa Fluor 488-conjugated goat anti-rabbit IgG and Texas Red-conjugated goat anti-mouse IgG secondary antibodies were purchased from Invitrogen Molecular Probes (Eugene, OR, USA). Horseradish peroxidase (HRP) conjugated to anti-mouse IgG and anti-rabbit IgG secondary antibodies were purchased from Cell Signaling Technology (Danvers, MA, USA). The Pierce BCA Protein Assay Kit was from Thermo Scientific (Waltham, MA, USA), and Novex precast Tris-Glycine gels were purchased from Invitrogen (Carlsbad, CA, USA) or Bio-Rad Laboratories (Hercules, CA, USA). Recombinant human Tumor Necrosis Factor-alpha (rhTNFα) was purchased from R & D Systems (Minneapolis, MN, USA). ProLong^®^ Gold Anti-fade Reagent containing 4′,6-diamidino-2-phenylindole (DAPI) for nuclear staining was obtained from Invitrogen (Carlsbad, CA, USA) and Life Technologies (Grand Island, NY, USA).

### 2.2. Cell Culture and Treatment

Human primary neonatal keratinocytes (HEKn) from single donors were purchased from Invitrogen Corp. (Carlsbad, CA, USA) and cultured in low calcium serum-free EpiLife medium supplemented with the Human Keratinocytes Growth Supplement Kit (HKGS Kit; Cat# S-001-K, Thermo Fisher Scientific, Rockford, IL, USA) along with gentamicin and amphotericin from GIBCO (Carlsbad, CA, USA). Cells were re-fed every 2–4 days and split as necessary. Cells were plated at 550,000 cells per 60-mm dish and treated with 1000× stocks of calcium chloride (dissolved in water) and/or the ligands of interest (Figure 2A). Rat osteosarcoma cells (UMR-106) were obtained from the American Type Culture Collection (Manassas, VA, USA) and maintained in Dulbecco’s Modified Eagles Medium (Hyclone, GE Healthcare, Logan, UT, USA) supplemented with gentamicin/amphotericin in a humidified atmosphere at 37 °C and 5% carbon dioxide. For immunofluorescence and Western blot analyses, cells were treated with different concentrations of agents at different time points (24–48 h) prior to harvest and analysis. In some experiments, cells were first pre-incubated or pre-treated for 48 h with respective ligands/agents and then treated with or without TNFα (15 ng/mL) for 30 min prior to harvest.

### 2.3. Morphology, Immunocytochemistry, and Immunofluorescence Analysis

Keratinocytes were seeded in four-chamber tissue culture glass slides and pre-treated with concentrations of each ligand as follows: curcumin (10–20 μM), 1,25D (0.1 μM), DHA (10–20 μM), and GW501516 (100–200 nM), for 24–48 h and processed as earlier described [28]. Briefly, after treatment with (or without) VDR ligands for 48 h, the cells were stimulated for 30 min before harvest with or without 15 ng/mL TNFα prior to being washed twice with 1× phosphate-buffered saline (PBS) (Ca^2+^/Mg^2+^-free). Subsequently, cells were fixed in 2% paraformaldehyde in a 1:1 mix of cold acetone/methanol in PBS for 20 min at room temperature, followed by 15 min at 4 °C, followed by three washes in 1× PBS. Cells were then permeabilized for 5 min at room temperature with Triton stabilization buffer (0.5% Triton X-100, 100 mM piperazine-N,N′-bis(2-ethanesulfonic acid (PIPES) buffer (K^+^-free), 4% PEG, and 1 mM ethyleneglycol-bis(aminoethylether)-tetraacetic acid (EGTA)) and washed three times in 1× PBS. Nonspecific epitopes were blocked with blocking solution (10% normal goat serum, 2.5% bovine serum albumin) in PBS for 20 min. Samples were blotted and subsequently incubated overnight at 4 °C with one of the following monoclonal or polyclonal primary antibodies diluted in blocking buffer: NR4A2/NURR1 (1:100 dilution), LCE3B–E (cross-reacts with four of the five LCE3 protein isoforms [27]) (1:100 dilution), Jun B (1:100 dilution), p-JNK (1:40 dilution), p-p38 (1:100 dilution), or filaggrin (1:50 dilution). Hereafter, the cells were washed three times and incubated with goat anti-rabbit IgG Alexa Fluor 488 or goat anti-mouse IgG-Texas Red secondary antibodies conjugated to HRP, all at 1:600 dilution in blocking buffer, for 45 min at 37 °C. Slides were washed twice in PBS for 10 min each followed by washing once in double-distilled water, and the cover slips were mounted on glass slides using ProLong^®^ Gold Anti-fade Reagent containing DAPI from Thermo Fisher Scientific (Cat# P36941, Rockford, IL, USA) for nuclear counter-staining. The mounted slides were allowed to cure overnight in the dark at room temperature. Automated images were acquired using the Nuance Imaging system with a camera equipped on a light microscope as described below.

### 2.4. Nuance Multispectral Imaging System FX—Software

Automated immunofluorescence images were acquired using an Olympus BX43 light microscope (Olympus America Inc., Center Valley, PA, USA) equipped with a CRI camera on a Nuance^TM^ Imaging FX system version 3.0.2 (Perkin Elmer, Inc., Waltham, MA, USA) using 20×/0.5 or 40×/0.75 objectives connected to a computer and an X-Cite^®^ Series 120 Q Sport light source. Data acquisition and image analysis using Nuance software technology plate-form were conducted as previously described [29,30]. Briefly, a spectral library was created using image cubes to define distinctive spectral curves for each fluorophore, and counterstained to adjust for background effects and to accurately quantify the positive staining of biomarkers using InForm version 1.4.0 software (Perkin Elmer Inc., Waltham, MA, USA), which allows for an objective analysis of biomarkers with increased accuracy. Isotype controls were used for immunostaining (proportion of green/red pixels for antigen staining) with values averaged from at least five fields for each slide sample.

### 2.5. PSORS4 Genotyping of HEKn Cell Lots

Genomic DNA was isolated from cells using a DNAeasy kit (Qiagen Corp., Valencia, CA, USA) according to the manufacturer’s protocol. A triple primer set was utilized to genotype cells: LCE3CF 5′-TCACCCTGGAACTAGACCTCA-3′; LCE3CR 5′-CTCCAACCACTTGTTCTTCTCA-3′; LCE3CR2D 5′-CATCCCAGGGATGCTGCATG-3′ [31]. PCR reactions contained approximately 130 ng/μL of HEKn genomic DNA from a single lot of cells, 0.5 μL of an 18 μM stock of the above three primers (0.9 μM final concentration of each primer), and 5 μL of Fast Start Universal SYBR Green Master Mix from Roche Applied Science (Indianapolis, IN, USA) in a total volume of 10 μL. An Applied Biosystems 2400 machine was programmed for 35 cycles: 94 °C for 30 s, 60 °C for 30 s, and 72 °C for 1 min, followed by a 72 °C step for 10 min. PCR products were resolved on 3% agarose gels. A single band at 199 bp indicates that cells harbor a homozygous *LCE3C_LCE3B* deletion (*LCE3C_LCE3B-del*), a single band at the position 240 bp indicates a homozygous intact locus, and the presence of both bands signifies a heterozygote.

### 2.6. Transient Transfection and Treatment of UMR-106 Cells

UMR-106 cells were plated at 650,000 cells/well in a 6-well plate. After 24 h of incubation, the cells were transfected with PolyJet reagent (SignaGen Laboratories, Gaithersburg, MD, USA) according to the manufacturer’s protocol. Briefly, each well received 20 μL/well PolyJet and 500 ng/well of pSG5-VDR M4, an expression plasmid containing the human VDR cDNA with translation starting at codon 4 (one of the common polymorphic variants of VDR [32]). After 24 h of incubation, the cells were treated with ethanol vehicle or 1, 10, or 100 nM 1,25D. RNA was harvested after 22–24 h of incubation.

### 2.7. Cell Harvesting and Total RNA Preparation

Cultured cells were harvested by trypsinization using standard techniques and cell pellets were washed with sterile phosphate-buffered saline. RNA isolation was performed using an Aurum Total RNA Mini Kit (Bio-Rad Corp., Hercules, CA, USA) from HEKn cells seeded at 550,000 cells per 60-mm plate and grown to a final confluency of approximately 65–70%. The quantity and purity of prepared RNAs were assessed by UV absorbance at 260 vs. 280 nm. Similar procedures were employed for the UMR-106 cells.

### 2.8. Primer Design and Testing

The UC Santa Cruz Genome Browser [33] was utilized to determine the coding sequence of the genes to be investigated. Unless otherwise referenced, Primer3Plus [34] was used to design primers that spanned an intro-exon junction. For the detection of human *LCE3* transcripts, the following primers were used: *LCE3A* forward primer 5′-CTGAGTCACCACAGATGCCG-3′ and reverse primer 5′-CTTGCTGACCACTTCCCCTG-3′; *LCE3B* forward primer 5′-CTCCTGCTGTGCTCCAAGAC-3′ and reverse primer 5′-ATCTTGCTGACCACTGCCTC-3′; *LCE3C* forward primer 5′-GGTCTGAGGGTTCTGTGCTC-3′ and reverse primer 5′-ACACTTGGGTGAGGGACAAC-3′; *LCE3D* forward primer 5′-CCCCAAAGAGCCCAGTACAG-3′ and reverse primer 5′-CTGTGGTGGTTCAGGAAGCA-3′; *LCE3E* forward primer 5′-CCCAAGTGTCCCCCAAAGAA-3′ and reverse primer 5′-CTGTGGTGGTTCAGGAAGCA-3′. For the detection of human *Jun B* and *NR4A2* transcripts, the following primers were used: human *Jun B* forward primer 5′-CGGCAGCTACTTTTCTGGTC-3′ and reverse primer 5′-GAAGAGGCGAGCTTGAGAGA-3′; human *NR4A2 (NURR1)* forward primer 5′-CTACGACGTCAAGCCACCTT-3′ and reverse primer 5′-TCATCTCCTCAGACTGGGGG-3′. Human glyceraldehyde 3-phosphate dehydrogenase (*GAPDH*) mRNA was amplified using forward primer 5′-TGACAACTTTGGTATCGTGGAAGG-3′ and reverse primer 5′-AGGGATGATGTTCTGGAGAGCC-3′. For the detection of rat transcripts, the following primers were used: rat *Nr4a2* forward primer 5′-CTACGCTTAGCATACAGGTC-3′ and reverse primer 5′-TTCCTTGAGCCCGTGTCT-3′ [35]. Rat *GAPDH* was amplified using forward primer 5′-AGGTCGGTGTGAACGGATTTG-3′ and reverse primer 5′-CATTCTCAGCCTTGACTGTGC-3′. All primer pairs were prepared as 18 μM stocks and stored at −20 °C.

### 2.9. Real-Time PCR

First strand cDNA was synthesized using a Bio-Rad iScript kit from total RNA isolated from HEKn cells. Quantitative real-time PCR was performed with Fast Start Universal SYBR Green Master Mix (Roche Applied Science) in an ABI 7500 Fast thermal cycler, or a BioRad CFX96 thermal cycler for data in Figure 7B only. Each GAPDH PCR well contained 0.5 μL of primers, 0.25 μL cDNA, and 5 μL of SYBR Green reagent mixed in a total volume of 10 μL. Wells for the detection of other gene products contained 0.5 μL of primers, 0.75 μL of cDNA, and 5 μL of SYBR Green. The temperature profile included 40 cycles with a melting step of 15 s at 95 °C and an annealing/elongation step of 1 min at 60 °C. Real-time PCR data were analyzed via the comparative C_t_ method and normalized to GAPDH. Fold effects for ligand treatments were calculated in relation to the samples treated with ethanol or DMSO vehicle.

### 2.10. Protein Extraction and Immunoblotting

After treatment with different concentrations of ligands at different time points, normal human keratinocyte cells were harvested and whole cell lysates were prepared for Western blot analysis. Briefly, cells were homogenized by sonication in ice-cold 1× RIPA lysis buffer (50 mM Tris-HCl, pH 7.4, 150 mM NaCl, 1 mM EGTA, 1 mM EDTA, 20 mM NaF, 100 mM Na_3_VO4, 0.5% NP-40, 1% Triton X-100, 1 mM PMSF) with freshly added protease inhibitor cocktail (Protease Inhibitor Cocktail Set III, Calbiochem, La Jolla, CA, USA). The homogenate was then centrifuged at 14,000× *g* for 25 min at 4 °C, and the supernatant was collected, aliquoted, and stored at −80 °C. For immunoblotting, 10–20 μg of protein was resolved on 8–12% SDS polyacrylamide (SDS-PAGE) gels and transferred onto nitrocellulose membranes. Blots were incubated in blocking buffer (5% non-fat dry milk/1% Tween 20; in 20 mM Tris-buffered saline (TBS), pH 7.6) for 45 min at room temperature, followed by incubation with a primary antibody directed against either LCE3B–E, Jun B, or NR4A2/NURR1 in blocking buffer overnight at 4 °C. Following several washes, membranes were incubated with the appropriate HRP-conjugated secondary antibody and detected by enhanced chemiluminescence (ECL) and autoradiography using a Bio-Rad Gel-Doc System (Bio-Rad Laboratories Inc., Hercules, CA, USA). Densitometric measurements of the bands were performed with image analysis software using the Biorad ChemiDoc MP imaging system (Bio-Rad, Hercules, CA, USA). To ensure equal protein loading, membranes were re-probed with antibodies to appropriate house-keeping proteins (GAPDH or vinculin) and processed as above. GAPDH or vinculin data were then used as normalization factors.

## 3. Results

To examine the effects of DHA and curcumin on human neonatal epidermal keratinocytes, we initially monitored the morphology of HEKn cells treated with different concentrations of ligands or vehicle control over time using phase contrast microscopy. As depicted in Figure 2B, we observed that these ligands differentially induced changes in cellular morphology reminiscent of keratinocyte differentiation.

We hypothesized that many of the effects of both DHA and curcumin on keratinocytes are mediated via the vitamin D receptor (VDR) acting to upregulate the expression of the *LCE3A/LCE3D/LCE3E* genes. First, we determined if the single-patient sample of HEKn cells harbored the *PSORS4* deletion and, if so, whether the deletion was homozygous or heterozygous, using a PCR protocol that incorporated a triple set of primers (see Methods Section 2.5). This reaction yielded a single PCR product at 199 bp (data not shown), indicative of a homozygous *LCE3C_LCE3B* deletion (*LCE3C_LCE3B-del*). Utilizing a homozygous deletant allowed for a direct test of the hypothesis that DHA and/or curcumin upregulate *LCE3A/LCE3D/LCE3E* mRNA expression without interfering background from PCR primers annealing to the similar *LCE3B* and/or *LCE3C* mRNAs.

**Figure 3:**
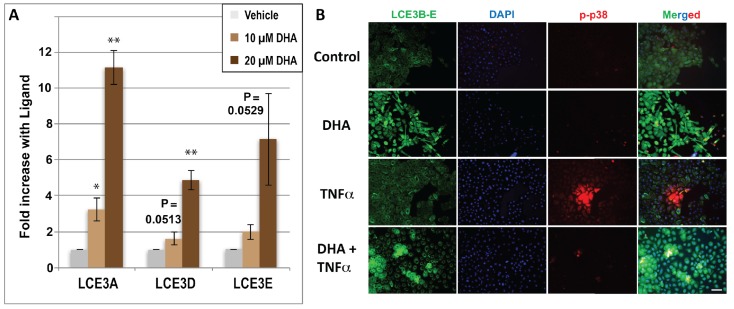
(**A**) Regulation by docosahexaenoic acid (DHA) of the *LCE3A, LCE3D* and *LCE3E* genes spared by the *PSORS4* deletion. Human primary neonatal keratinocytes (HEKn) cells homozygous for the deletion were treated with ethanol vehicle (negative control) or DHA (10 or 20 μM), and total RNA was prepared and analyzed for the expression of mRNA using specific primers for each LCE3 isoform. Bar graphs represent the average of three independent experiments ± STDEV. Asterisks denote results with ligands that are significantly different from ethanol controls as determined by the Student’s two-tailed *t*-test: * *p* < 0.05; ** *p* < 0.01. *p*-Values approaching 0.05 are given above the corresponding bar. (**B**) Regulation of LCE3 proteins (and p-p38) by DHA and tumor necrosis factor-alpha (TNFα) as monitored by immunohistochemistry using an antibody that cross-reacts with LCE3B, LCE3C, LCE3D, and LCE3E proteins. Cells were cultured in four chamber tissue culture glass slides and treated for 48 h with (10 μM) or without DHA as indicated. Treatment with TNFα (15 ng/mL) occurred 30 min prior to fixing and binding of permeabilized cells to the indicated antibodies. Image acquisition is described in Methods and representative images are shown. Images in right-hand column represent a merging of all three signals (LCE3B–3E antibody (green), 4′,6-diamidino-2-phenyl (DAPI) (blue), and p-p38 antibody (red)) at a magnification of ×200.

The first experiment was performed by treating HEKn cells homozygous for *LCE3C_LCE3B-del* with two concentrations of DHA. The selection of concentrations was based on a previously published competition binding assay [15] which indicated that DHA competes with radioactively labeled 1,25D for VDR binding with an IC_50_ of approximately 10 μM. As shown in Figure 3A, DHA indeed upregulates *LCE3* mRNA expression in a dose-dependent manner, with 20 μM DHA eliciting 11-fold, 5.5-fold, and 7-fold increases in mRNAs for *LCE3A, LCE3D,* and *LCE3E,* respectively. These mRNA effects are superior to the respective 3.6-fold, 4.5-fold, and 4.7-fold increases in mRNAs for *LCE3A, LCE3D,* and *LCE3E* reported previously by our group for HEKn cells treated with 100 nM 1,25D [11], which serves as a published positive control (employing the hormonal VDR ligand) for the present experiments. To confirm this effect of DHA at the protein level, cells were treated in the presence or absence of single doses of DHA (10 μM) with or without activation by recombinant human TNFα. Protein expression of *LCE3* gene products was monitored by double immunofluorescent microscopy as described in the Methods section; the antibody for LCE3 expression recognizes LCE3B, LCE3C, LCE3D, and LCE3E. As shown in Figure 3B, treatment with 10 μM DHA alone for 48 h strongly induced the expression of LCE3 proteins compared to vehicle control cells (see LCE3B–E column, comparing top two panels), with no discernible effect on the levels of phosphorylated p38 MAP kinase (p-p38). Phospho-p38 was included in the study because it is a pro-inflammatory factor that is activated in psoriasis [36]. Because the pro-inflammatory cytokine TNFα is critically involved in the early phase of psoriasis [37], we next examined the effect of TNFα activation on the response to DHA ligands. As shown in Figure 3B, treatment with TNFα did not discernibly modulate LCE3B–E protein expression, whether in the presence or absence of DHA (LCE3B–E column, compare bottom two panels with top two panels), but strongly induced the phosphorylation of p38 (see p-p38 column, second panel from the bottom). Additionally, the pre-treatment of keratinocytes with DHA for 48 h, prior to activation with TNFα for 30 min, appeared to block the effect of TNFα on p38 phosphorylation (Figure 3B; p-p38 column, bottom panel). A subset of these results was further corroborated by Western blot analyses (see last section of Results below).

**Figure 4:**
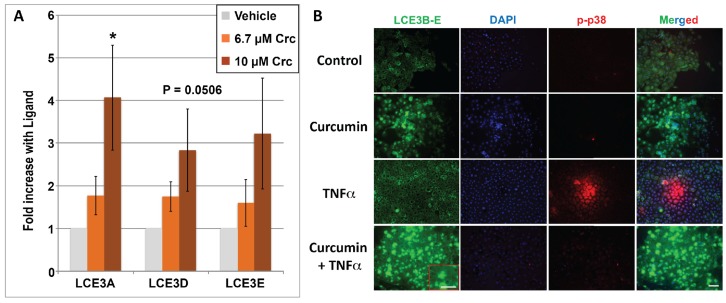
(**A**) Regulation of *LCE3A, LCE3D*, and *LCE3E* mRNA by curcumin (Crc). HEKn cells were treated with ETOH vehicle (negative control) or curcumin (at 6.7 or 10 μM concentration). Bar graphs show real-time PCR results, which are the average of four independent experiments ± STDEV. An asterisk denotes ligand-treated averages that are significantly different from ethanol controls as determined by two-tailed Student’s *t*-test, * *p* < 0.05. One of the four replicates of this experiment was performed with HEKn cells heterozygous for the *PSORS4* deletion, which yielded results very similar to the three replicates from homozygous cells. (**B**) Regulation of LCE3 and p-p38 proteins by curcumin and TNFα as monitored by immunohistochemistry. Cells were cultured in four chamber tissue culture glass slides and treated for 48 h with (15 μM) or without curcumin as indicated. Treatment with TNFα (15 ng/mL) occurred 30 min prior to fixing and binding of permeabilized cells to the indicated antibodies. Image acquisition is described in Methods and representative images are shown. Images in right-hand column represent a merging of all three signals (LCE3B-3E (green), DAPI (blue), and p-p38 antibody (red)) at a magnification of ×200. Insets in red boxes indicate a higher power magnification (×400) of the selected section.

A second dietary agent, namely curcumin, a turmeric derivative and bioactive polyphenol, was similarly evaluated in cultured keratinocytes. Because curcumin exhibits a comparable VDR competition profile to DHA (an IC_50_ of approximately 5–10 μM [15]), and has been reported to be a bona fide VDR ligand [38] with beneficial effects on skin repair [24], we treated HEKn cells with concentrations of curcumin similar to those used for DHA, namely 6.7 and 10 μM for mRNA studies and 5, 10, and 20 μM for immunocytochemistry. Also, DMSO rather than ethanol was employed as the solvent vehicle for curcumin. The results in Figure 4A reveal that curcumin is capable of upregulating *LCE3A*, *LCE3D*, and *LCE3E* mRNAs 3- to 4-fold in a dose-dependent manner at concentrations corresponding closely to the concentrations capable of competing with 1,25D for binding to the VDR. This magnitude of induction of *LCE3* genes is quite comparable to that of 3.6- to 4.7-fold achieved by 100 nM 1,25D as previously published [12]. Immunostaining for protein expression in human keratinocytes confirmed that curcumin treatment alone strongly induced the protein expression of LCE3B–E (Figure 4B; LCE3B–E column, compare top two panels). Analogous to the DHA study shown in Figure 3B, a 30-min treatment with TNFα (±curcumin) was also included (lower two rows of Figure 4B), and there is a suggestion that the combination of curcumin and TNFα yields a higher induction of LCE3B–E protein expression than curcumin alone (Figure 3B, LCE3D–E column, compare second and fourth panels). Finally, the phosphorylation of MAPK p38 in response to the various treatments was also included, with curcumin showing effects similar to DHA, namely an ability to block p38 phosphorylation in response to TNFα (Figure 4B, p-p38 column, compare third and fourth panels). A subset of these results was further corroborated by Western blot analyses (see last section of Results).

Although the present data are consistent with the conclusion that DHA is acting as a VDR ligand, literature reports indicate that DHA effects in skin may be mediated by a peroxisome proliferator-activated receptor (PPAR), presumably PPARδ, which is the predominant isoform expressed in skin [26]. We and others have recently shown that PPARδ is overexpressed in human psoriatic as well as in murine psoriasis-like skin lesions [39], and that treatment with delphinidin, another VDR ligand, normalized the expression in a preclinical mouse model of psoriasis [30]. To determine whether PPARδ is capable of upregulating LCE3 mRNAs in human keratinocytes, a selective ligand for PPARδ (GW501516) was examined at a single 100 nM concentration which, according to published reports, represents a saturating dose for activating PPARδ [40]. The results (data not shown) indicated a nonsignificant trend toward a very slight (<1.5-fold) upregulation of *LCE3A*, *LCE3D*, and *LCE3E* mRNA expression. Moreover, Western blot data from keratinocytes treated for 24 to 48 h with two doses of GW501516 (100 and 200 nM) yielded no significant increase in the expression of LCE3 proteins (see last section of Results). In this same experiment, 10–20 μM DHA or curcumin elicited a statistically significant enhancement in LCE3 protein levels, providing a positive control and indicating that liganded PPARδ is not an inducer of LCE3 protein in HEKn cells. Based on these data, we conclude that liganded PPARδ plays little or no role in *LCE3* gene expression, and this result allows us to distinguish the relative contributions of VDR and PPARδ in mediating the actions of DHA with respect to LCE3 maintenance. The conclusion is that VDR alone executes the function of DHA to induce *LCE3* gene and protein expression. This does not eliminate potential cross-talk between the signaling of the VDR and PPAR systems in skin, at least with regard to the regulation of genes other than those of the LCE3 class. For example, GW501516 is a strong inducer of NR4A2 protein in HEKn cells, as are 1,25D and curcumin (see last part of Results).

**Figure 5:**
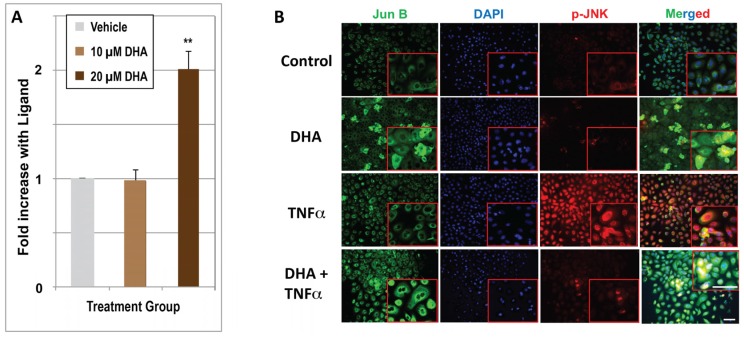
(**A**) Regulation of *Jun B* mRNA by DHA. HEKn cells were treated with ethanol vehicle (control), or with either 10 μM DHA or 20 μM DHA. RNA isolation, synthesis of first strand DNA, and real-time PCR are described in Methods. Results are means from three independent experiments ± STDEV. The double asterisk (**) denotes an average that is statistically significant by Student’s *t*-test from the ethanol control, *p* < 0.01. (**B**) Regulation of Jun B and p-JNK proteins by DHA and TNFα as monitored by immunohistochemistry. Cells were cultured in four chamber tissue culture glass slides and treated for 48 h with (10 μM) or without DHA as indicated. Treatment with TNFα (15 ng/mL) occurred 30 min prior to fixing and binding of immobilized proteins to the indicated antibodies. Image acquisition is described in Methods and representative images are shown. Images in the right-hand column represent a merging of all three signals (Jun B antibody, DAPI, and p-JNK antibody), measured using Nuance software as described in Methods. (Magnification ×200). Insets in red boxes indicate a higher power magnification (×400) of the selected section. The yellow color indicates co-localization of both markers.

We next assessed whether DHA upregulates *Jun B*, for which depressed expression or gene deletion has been noted to be associated with psoriasis. Quantitative real-time PCR results (Figure 5A) indicate that DHA significantly upregulates *Jun B* mRNA expression, but the effect is minimal (2-fold) after 24 h of treatment, and is not evident until DHA is present at the higher 20 μM concentration. Nevertheless, we previously demonstrated *Jun B* induction by 1,25D in KERTr human keratinocytes, utilizing microarray technology to quantitate mRNA [8]. In the present report, utilizing immunofluorescence to determine the effects of 48 h of DHA treatment, we observed an increase in Jun B protein expression (Figure 5B) in both the presence and absence of TNFα treatment. The immunofluorescence experiment also included the monitoring of JNK phosphorylation using a specific phospho-JNK antibody (see p-JNK column in Figure 5B). As expected, JNK is phosphorylated in response to 30 min of treatment with TNFα, an effect that is largely abolished when TNFα-treated cells are pretreated with DHA (Figure 5B, p-JNK column, compare bottom two panels). The upregulation of Jun B by DHA was confirmed by Western blot analysis (see last section of Results), exhibiting a significant, dose-dependent upregulation of Jun B protein at both 24 and 48 h, approaching the dramatic action of 100 nM 1,25D to enhance Jun B protein levels.

**Figure 6:**
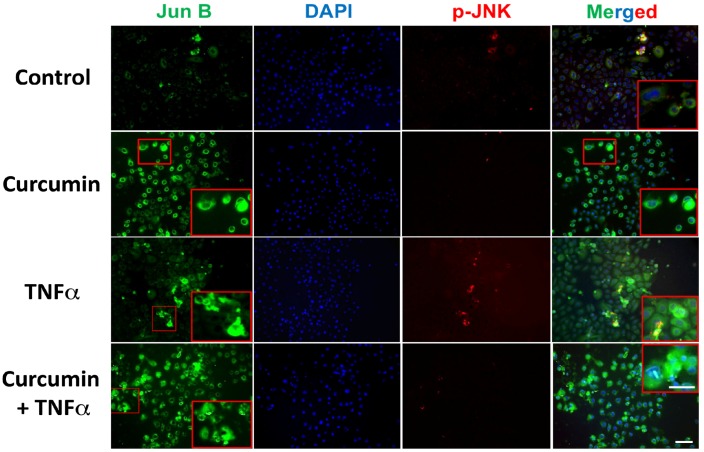
Regulation of Jun B and p-JNK proteins by curcumin and TNFα as monitored by immunohistochemistry. Cells were cultured in four chamber tissue culture glass slides and treated for 48 h with or without curcumin (15 μM) as indicated. Treatment with TNFα (15 ng/mL) occurred 30 min prior to fixing in 2% paraformaldehyde and binding of immobilized proteins to the indicated antibodies. Image acquisition is described in Methods and representative images are shown. Images in right-hand column represent a merging of all three signals (Jun B antibody, DAPI and p-JNK antibody), measured using Nuance software as described in Methods. (Magnification ×200). Insets in red boxes indicate a higher power magnification (×400) of the selected section.

The ability of curcumin ± TNFα to upregulate Jun B expression was also investigated, and Figure 6 illustrates the immunofluorescence results using an anti-Jun B antibody. Treatment with curcumin increased levels of Jun B (Figure 6, Jun B column, compare top two panels), an effect that was confirmed with Western blotting (Figure 8B), which demonstrated a significant and dose-dependent increase in Jun B protein after both 24 and 48 h of treatment using doses of 5, 10, and 20 μM. Curcumin also appears to dampen the Jun B-induced phosphorylation of p-JNK by TNFα (Figure 6, p-JNK column, compare bottom two panels). Thus, two low-affinity VDR ligands (DHA and curcumin) evaluated in the present study are able to induce Jun B, as well as to oppose TNFα-induced p-JNK phosphorylation.

**Figure 7:**
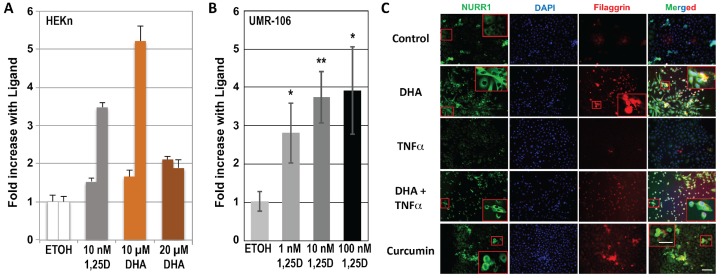
Regulation of *NURR1* (*NR4A2*) mRNA by 1,25D. (**A**) Response of *NURR1* mRNA to 1,25D and DHA in HEKn cells. Cells were plated as described in Methods and dosed with the indicated concentrations of 1,25D or DHA for 22–24 h. Total RNA and first strand cDNA were then prepared, and real-time qPCR was performed using primers to human *NURR1* as described in Methods. Error bars represent STDEV of triplicate real-time PCR wells from each of two independent experiments. (**B**) A similar experiment to (**A**), but performed using increasing doses of 1,25D in rat UMR-106 cultures. Results are means of three independent experiments ± STDEV, * *p* < 0.05, ** *p* < 0.01 compared to control by Student’s *t*-test. (**C**) Regulation of NURR1 and filaggrin proteins by DHA and TNFα as monitored by immunohistochemistry. Cells were cultured in tissue culture glass slides and treated for 48 h with (10 μM) or without DHA as indicated. Treatment with TNFα (15 ng/mL) occurred 30 min prior to fixing in 2% paraformaldehyde and binding of immobilized proteins to the indicated antibodies. Image acquisition is described in Methods and representative images are shown. Images in right-hand column represent a merging of all three signals (NURR1 antibody, DAPI and filaggrin antibody), measured using Nuance software as described in Methods. (Magnification ×200). Insets in red boxes indicate a high power magnification (×400) of the highlighted section.

**Figure 8:**
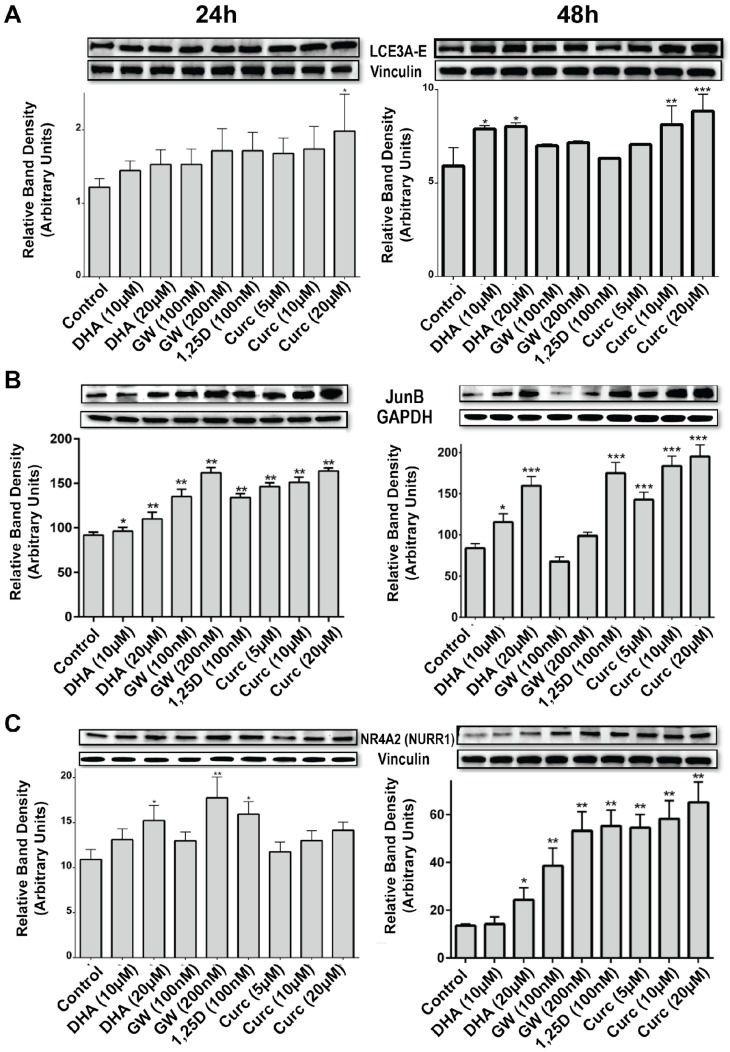
Dose-dependency of protein expression in response to 1,25D, curcumin (Curc) (ligand for the vitamin D receptor), GW501516 (GW; ligand for PPARδ), and DHA (ligand for both receptors). (**A**) Protein expression for LCE3 proteins as monitored by an antibody that recognizes four LCE3 isoforms (LCE3B/LCE3C/LCE3D/LCE3E), using Vinculin expression as an unregulated control to normalize for protein loading. (**B**) Protein expression of Jun B, using GAPDH as an unregulated control. (**C**) Protein expression of NR4A2 (NURR1), using Vinculin as an unregulated control. All bars represent an average of at least five fields for each sample (blots were normalized using Chemidoc quantification analysis software as described in Methods) ±STDEV. * *p* < 0.5, ** *p* < 0.01, *** *p* < 0.001.

Finally, we explored the possibility that VDR ligands, including 1,25D, DHA, and curcumin, regulate the expression of the nuclear receptor NURR1, also known as NR4A2, pursuing a notion previously derived conceptually from the recent demonstration that some VDR actions follow a secondary induction mechanism, whereby liganded VDR first induces NURR1, which in turn activates the expression of the gene(s) of interest [25]. Moreover, as noted above, the LCE3 VDRE shown in Figure 1 contains within its 5′ half-element a consensus binding sequence for the NR4A2 monomer, caGGGTGA. For these reasons, we examined the level of NURR1 transcripts in DHA-, 1,25D-, and vehicle-treated HEKn cells. The results (Figure 7A) demonstrate that both 1,25D and DHA upregulate *NURR1* mRNA in HEKn cells, although there exists no indication of a classic dose-response relationship for DHA, and the results are variable for treatment with 1,25D and 10 μM DHA. To definitively demonstrate that *NURR1* can be induced in a VDR and ligand dose-dependent fashion, UMR-106 osteoblast-like cells were transfected with human VDR and treated with concentrations of 1,25D from 1 to 100 nM. Data for *NURR1* mRNA levels compared with vehicle controls, as displayed in Figure 7B, exhibit clear 1,25D dose-dependency for a 3- to 4-fold induction of *NURR1* by the hormonal vitamin D. We followed up these mRNA studies by investigating the effect of a single dose of DHA or curcumin treatment using both immunofluorescence as well as Western blotting on the protein expression of NURR1 as well as filaggrin, another biomarker of skin inflammatory disease. Immunofluorescence after 48 h of treatment with ligands reveals a striking ability of both DHA alone and curcumin alone to upregulate both NURR1 and filaggrin (see NURR1 and filaggrin columns in Figure 7C, comparing second and fourth panels to top control panel). These results were confirmed by Western blotting (Figure 8C). Although the DHA effects pictured in Figure 8C are statistically significant only at the higher (20 μM) dose, effects with both DHA and curcumin displayed a dose-response at both time points. Importantly, 1,25D exerts a dramatic positive induction of NURR1 protein in HEKn cells (Figure 8C). Taken together, these results are consistent with the intermediary function of NURR1 as a secondary mediator of at least part of the action of VDR ligands in osteoblasts and skin, with putative occupation of the NURR1 site embedded within the VDRE identified in the LCE gene region being of potential mechanistic significance.

## 4. Discussion and Conclusions

Analogs of 1,25D are used routinely for psoriasis treatment, often in combination with an anti-inflammatory steroid [41]. Previous studies from our laboratory have pursued the hypothesis that 1,25D and other VDR ligands improve symptoms of psoriasis by upregulating skin repair genes, and in two recent publications by our group, we reported that the VDR ligands 1,25D, delphinidin, and cyanidin upregulate *LCE3A*/*LCE3D*/*LCE3E* gene expression, potentially to compensate for the common genomic deletion of *LCE3B* and *LCE3C* [11,12].

The dietary lipids DHA (an omega-3 fatty acid) and curcumin (a bioactive component of turmeric spice) were selected for investigation as both have been subjects of clinical trials for the treatment of psoriasis (NCT01351805 and NCT00235625). In addition to these clinical trials, dietary studies have concluded that psoriatic patients typically consume diets low in omega-3 fatty acids, including DHA [42], and conversely, that a diet low in calories but high in omega-3 fatty acids appears to confer a better response to immunomodulatory drugs in a cohort of obese patients with plaque-type psoriasis [43]. Another dietary study found that adherence to a Mediterranean diet was inversely related to psoriasis area severity index (PASI) score, and that high consumption of fish (which contain omega-3 fatty acids including DHA) was independently negatively associated with a high PASI score [44]. Although it is difficult to separate the potential effects of DHA from those of other dietary components, these studies, taken together, suggest that consumption of omega-3 fatty acids, including DHA, may represent a useful adjuvant to other psoriasis therapies.

In addition to other modes of action, the current study pursues the notion that DHA and curcumin may act as VDR ligands to regulate genes involved in psoriasis pathology. Both DHA and curcumin have been shown, like other alternative, low-affinity VDR ligands such as delphinidin and cyanidin, to compete with 1,25D for direct, low-affinity binding to VDR [15]. The data shown in Figure 3 indicate that DHA significantly upregulates *LCE3* gene expression in cells homozygous for *LCE3C_LCE3B-del*. We propose that this induction of *LCE3* gene products compensates for the loss of *LCE3B* and *LCE3C* in skin repair, under the assumption that the highly similar *LCE3* gene products are functionally redundant [27]. Curcumin was also demonstrated to be capable of upregulating *LCE3A*, *LCE3D*, and *LCE3E* genes (Figure 4). Given that the effective concentrations of DHA and curcumin employed in these experiments coincide with those that competitively bind to VDR, we postulate that these ligands upregulate *LCE3* gene expression, at least in part, by binding to, and activating the VDR-RXR heterodimer. Our previous work identified a VDRE adjacent to the *LCE3A* gene (Figure 1) that is capable of conferring regulation onto a heterologous reporter gene by 1,25D, delphinidin, or cyanidin in transfected CCD-1106 KERTr human keratinocyte cells [11], lending further support to the notion that liganded VDR may induce the expression of these genes in vivo.

Recent reports reveal that the presence of *LCE3C_LCE3B-del* within the *LCE3* gene cluster has a lengthy evolutionary history in the hominid lineage. The deletion appears to have arisen in the common ancestor to modern humans and Denisovans [45,46]. Based on an analysis of genomic DNA from ancient hominins as well as modern humans, including a haplotype analysis of the deletion as well as flanking regions in the *LCE3* locus, the authors of these studies conclude that the 32 kb *LCE3C_LCE3B* deletion has been maintained under balancing selection in the human lineage. In considering the potential reasons as to why both LCE3 haplotypes are currently found in all tested human populations, including those from Eurasia, Africa, and the Americas [46,47], these authors quoted Bergboer et al. [48], who hypothesized that *LCE3C_LCE3B-del*, by delaying skin repair, could allow for greater penetration of microbial antigens, which then serve as a natural “vaccine” against future infections (while also posing an increased risk for an autoimmune response). Further, the hypothesis explaining the retention of the intact *LCE3* cluster is that the full complement of five *LCE3* genes confers a superior ability to support skin repair. Whatever the exact explanation, it appears that an interplay between these two sets of advantages/disadvantages has led to the preservation of both alleles in a state of balance over hundreds of thousands of years.

The extent to which DHA upregulates LCE3 mRNA expression exceeds 10-fold in the case of *LCE3A*, which is noticeably greater than the approximate 4-fold achieved by 10^−8^ M 1,25D in prior studies [11,12]. This comparison suggests that, at least for the upregulation of *LCE3* gene expression, DHA may be superior to the currently used therapeutic agent 1,25D (or analogs thereof), taking into account that higher concentrations of DHA are required [19] to achieve this effect due to the lower affinity of DHA for the receptor. This result positions both DHA and the previously tested cyanidin compound [11] as two dietary, non-toxic nutrients that are each capable of upregulating the expression of *LCE3* skin repair genes in a fashion superior to that of 1,25D, the currently used anti-psoriatic agent. Moreover, curcumin is yet another candidate alternative to 1,25D chemical analogs for psoriasis treatment, as its 3- to 4-fold action to induce *LCE3A* mRNA (Figure 4A and Figure 8A) is equivalent in magnitude to the effect of 100 nM 1,25D, but curcumin is less prone than 1,25D to induce toxic hypercalcemia and likely is endowed with beneficial influences not intrinsic to 1,25D.

The results presented herein (Figure 3, Figure 4 and Figure 5) do not rule out the possibility that DHA, curcumin, or other VDR ligands may act on LCE3 gene transcription via additional mechanisms. Indeed, literature reports have suggested that DHA effects in the skin are mediated, not by VDR, but rather via peroxisome proliferator-activated receptor (PPAR) isoforms such as PPARδ [26]. Furthermore, the doses of DHA that are capable of activating PPARδ are similar to doses necessary for the activation of VDR [49]. By observing that treatment with a saturating concentration of the PPARδ ligand GW501516 only modestly (and not statistically significantly) upregulated *LCE3A*, *LCE3D*, and *LCE3E* mRNAs (data not shown), and did not significantly enhance LCE3 protein expression (Figure 8A), we conclude that the effect of DHA on *LCE3* gene expression is exerted predominantly via the activation of VDR. Thus, DHA appears to function via a mechanism involving the association of liganded VDR with VDREs in target genes. To prove conclusively that liganded VDR docks on the postulated LCE3 gene region VDRE (Figure 1) will require in vivo ChIP-seq experiments in skin, a technical challenge which is beyond the scope of the current investigation, but is warranted for future studies.

The involvement of TNFα in the pathogenesis of psoriasis is well documented [50] and is the target of biological therapies that have proven effectiveness in severe cases [51]. The involvement of the MAP kinase p38 in the pathogenesis of psoriasis and its activation by the TNFα pathway are also well established [52]. We therefore investigated whether the VDR ligands DHA and curcumin could impact p38 phosphorylation in response to TNFα treatment of keratinocytes. Our finding that both ligands inhibit TNFα-induced p38 phosphorylation (Figure 3B and Figure 4B) suggests that these VDR ligands have anti-psoriatic actions besides the induction of skin repair involving *LCE3* gene products. The effects of both ligands on TNFα signaling are similar to those observed in the imiquimod-induced mouse models of psoriasis by curcumin [53], and in other tissues, such as rat endothelial cells, by DHA [54]. In the case of DHA, the involvement of PPAR receptors such as PPARγ in these effects cannot be excluded (see [55]). Similarly, the effect of TNFα on JNK phosphorylation is also relevant to psoriasis [56]. Again, the pretreatment of keratinocytes with either DHA or curcumin inhibited the TNFα-induced phosphorylation of JNK (Figure 5B and Figure 6), another indication that the anti-psoriatic mechanisms of these ligands extend to the TNFα pathway, which is successfully targeted by biologic treatments for moderate to severe psoriasis.

As a concluding experiment in this study, we evaluated whether VDR ligands, including DHA, 1,25D, and curcumin, regulate the expression of the nuclear receptor NURR1, also known as NR4A2, based on recent insight that specific VDR actions appear to involve a secondary mechanism whereby liganded VDR upregulates NURR1 expression, which in turn activates the expression the gene(s) of interest [25]. Moreover, increased expression of *NURR1* mRNA and protein occurs in involved psoriasis skin compared with uninvolved and normal skin [57], which is consistent with another recent report showing that NURR1 expression is crucial for the development of mature, fully functional Th17 cells [58] that have, in turn, been shown to play an important role in psoriasis pathogenesis via the production of IL-17 and IL-23 cytokines [59]. For these reasons, it was of interest to investigate the potential ability of DHA, curcumin, and 1,25D to control NURR1 expression. The results (Figure 7 and Figure 8C) revealed that DHA, curcumin, and 1,25D induce, rather than repress, NURR1 expression, consistent with the hypothesis that NURR1 acts as a secondary mediator of at least part of the function of VDR ligands to induce skin repair genes such as the LCEs. However, because NURR1 is apparently a pro-psoriatic transcription factor in human skin [57], its induction by VDR ligands complicates our understanding of the mechanism whereby vitamin D analogs are effective in suppressing mild to moderate psoriasis symptoms. In other words, by inducing NURR1, VDR ligands could conceivably aggravate inflammation and proliferation as collateral effects to inducing skin repair genes, although one could argue that NURR1 is actually a trigger for normal skin remodeling in place of psoriatic pathology. It is also possible that the relative timing of NURR1 upregulation might be important with respect to its effects on inflammation and/or proliferation versus skin repair, an issue that could be addressed in future studies. In conclusion, identified herein is a secondary induction mechanism whereby VDR ligands increase the expression of NURR1, which in turn may function as the primary inducer of skin repair genes such as the LCE3 ensemble. Again, proof of this concept will require in vivo ChIP-seq experiments in skin, determining whether NURR1 is the transcription factor actually docked on the composite response element for VDR and NURR1 identified in the LCE3 gene region (Figure 1).

Given the lack of understanding of how 1,25D analogs improve symptoms of psoriasis, the effect of these compounds, as well as low-affinity VDR ligands such as DHA, curcumin, cyanidin, delphinidin, and others, could be examined on the expression of genes harbored in different loci. One of many possible examples is secreted mammalian Ly6/urokinase plasminogen activator receptor-related protein (SLURP-2), a gene that is strongly induced in psoriatic skin lesions [60]. Finally, since 14,21-dihydroxy-DHA has been shown to be a dramatic wound healing lipid in murine models [61], it is tempting to speculate that cellular metabolites of DHA, and possibly other low-affinity VDR ligands, could be discovered as high-affinity nuclear receptor ligands. These novel super-bioactive metabolites, if identified, could prove vastly more efficacious than their low-affinity VDR ligand precursors or even than 1,25D itself. In conclusion, the present investigation reveals the ability of nutrient or diet-derived VDR ligands to upregulate specific skin-expressed genes and proteins with relevance for psoriasis, and sets the stage for future studies examining the regulation of psoriasis-related skin-expressed genes by novel metabolites of low-affinity VDR ligands such as DHA and curcumin, potentially leading to drug discovery of new molecular-based treatments for mild/moderate psoriasis. Further studies beyond the scope of the current manuscript are warranted to critically examine these observations and to decipher their detailed molecular mechanisms.
